# Interactions between DMPC Model Membranes, the Drug Naproxen, and the Saponin *β*-Aescin

**DOI:** 10.3390/pharmaceutics15020379

**Published:** 2023-01-22

**Authors:** Pia Hägerbäumer, Friederike Gräbitz-Bräuer, Marco Annegarn, Carina Dargel, Tim Julian Stank, Thomas Bizien, Thomas Hellweg

**Affiliations:** 1 Physical and Biophysical Chemistry, Bielefeld University, Universitätsstr. 25, 33615 Bielefeld, Germany; 2 Institute of Physical Chemistry, University of Münster, Corrensstr. 28/30, 48149 Münster, Germany; 3 Synchrotron SOLEIL, L’Orme des Merisiers, CEDEX, 91190 Saint-Aubin, France

**Keywords:** DMPC, small unilamellar vesicles (SUVs), nonsteroidal anti-inflammatory drug, naproxen, saponin, β-aescin, SAXS, WAXS, DSC, PCS

## Abstract

In this study, the interplay among the phospholipid 1,2-dimyristoyl-*sn*-glycero-3-phosphocholine (DMPC) as a model membrane, the nonsteroidal anti-inflammatory drug naproxen, and the saponin β-aescin are investigated. The naproxen amount was fixed to 10 mol%, and the saponin amount varies from 0.0 to 1.0 mol%. Both substances are common ingredients in pharmaceutics; therefore, it is important to obtain deeper knowledge of their impact on lipid membranes. The size and properties of the DMPC model membrane upon naproxen and aescin addition were characterized with differential scanning calorimetry (DSC), small- and wide-angle X-ray scattering (SAXS, WAXS), and photon correlation spectroscopy (PCS) in a temperature-dependent study. The interaction of all substances was dependent on the lipid phase state, which itself depends on the lipid’s main phase transition temperature $T_{m}$. The incorporation of naproxen and aescin distorted the lipid membrane structure and lowers $T_{m}$. Below $T_{m}$, the DMPC–naproxen–aescin mixtures showed a vesicle structure, and the insertion of naproxen and aescin influenced neither the lipid chain–chain correlation distance nor the membrane thickness. Above $T_{m}$, the insertion of both molecules instead induced the formation of correlated bilayers and a decrease in the chain–chain correlation distance. The presented data clearly confirm the interaction of naproxen and aescin with DMPC model membranes. Moreover, the incorporation of both additives into the model membranes is evidenced.

## 1. Introduction

One of the main components of biomembranes are phospholipids, which are amphiphilic molecules that consist of a substituted phosphate group as the hydrophilic head, and two hydrocarbon chains as the hydrophobic tail [1,2,3]. Because of their structure, phospholipids can be used for the production of biological model membranes. These are particularly useful in studying the interaction of biomolecules and drugs with the lipids forming the membrane [4,5]. In aqueous solution, phospholipids tend to assemble into spherical compartments, so-called vesicles that comprise one or more lipid bilayers [6]. Via extrusion through a porous membrane or sonication, small unilamellar vesicles (SUVs) are obtained [7,8]. SUVs can, for instance, be used for medical applications such as drug delivery [9,10,11].

**Figure 1:**
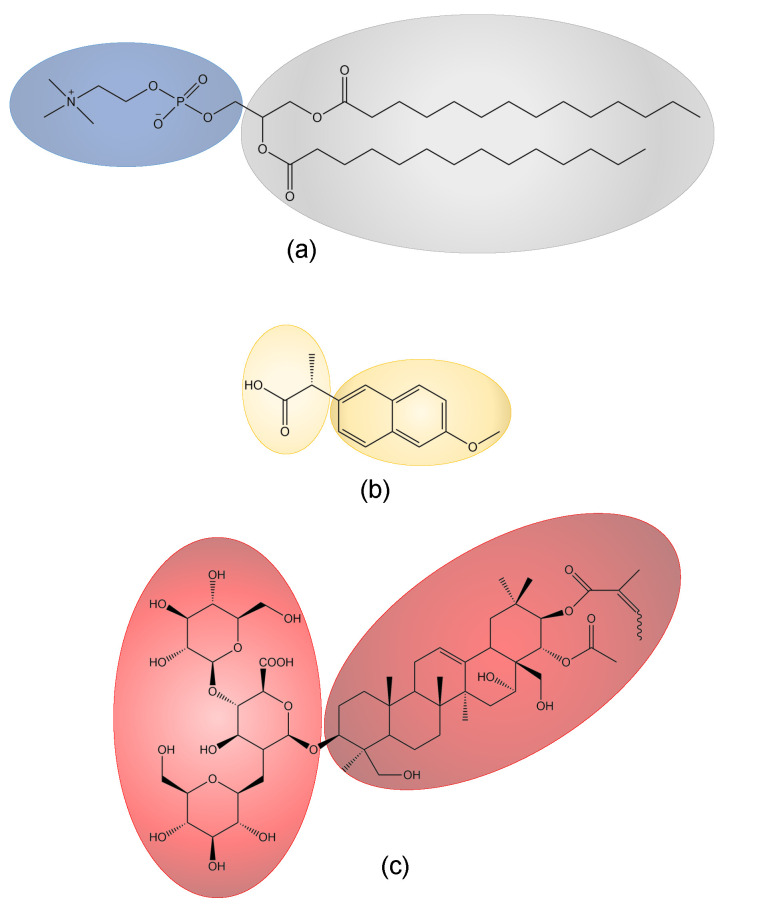
Chemical structures of (**a**) phospholipid DMPC, (**b**) NSAID naproxen, and (**c**) saponin β-aescin.

Phospholipid membranes show interesting temperature-dependent phase behavior [12]. An often-studied phospholipid is the synthetically produced 1,2-dimyristoyl-*sn*-glycero-3-phosphocholine (DMPC), which posses a phosphatidylcholine unit as the hydrophilic and two tetradecane fatty acid esters as the hydrophobic part [3]. Its molecular structure is shown in Figure 1a. At low temperatures, the DMPC membrane is present in a gel-like L${}_{\beta^{\prime }}$ phase in which the lipid molecules are ordered in an *all-trans* configuration. At 13.7 ${}^{{}^{\circ}}C$, the lipid molecules rearrange in a prephase transition into the P${}_{\beta^{\prime }}$ phase, where the molecules are ordered hexagonally. The main phase transition temperature $T_{m}$ of DMPC is reached at 24 ${}^{{}^{\circ}}C$; above this temperature, the membrane adopts a liquid crystalline phase L${}_{\alpha }$ where the molecules are arranged in gauche configuration [13].

Interaction with other substances or even their intercalation into the membrane can significantly modify the membrane’s properties. Nonsteroidal anti-inflammatory drugs (NSAIDs) are therapeutic drugs that inhibit the cyclooxygenase pathway; hence, they are used against fever, inflammation, and pain [14,15,16]. Common representatives are naproxen, ibuprofen, and diclofenac [17], which all interact with phospholipid membranes [15,16,18,19,20,21]. In this study, naproxen (Figure 1b) is used as representative of this substance class and in comparison to the prior investigated ibuprofen [10,22,23].

A study by Lichtenberger et al. states that NSAIDs bind to zwitterionic phospholipids via both hydrophobic and electrostatic interactions. The latter are established by the positively charged nitrogen of the phospholipid (see Figure 1a) and the carboxyl group of the NSAID, which is normally negatively charged [24]. Due to these interactions and the related perturbation of the regular lipid membrane through the integration of the NSAID, the $T_{m}$ of these membranes is lowered [15,22]. Moreover, membrane fluidity can be affected by the integration of the NSAID into the lipid membrane [20].

Saponins are natural biosurfactants and can be isolated from plants [25]. Their name originates from the Latin word “sapo”, meaning “soap”, and is related to their foaming properties and their use as washing agents. They can be classified by their molecular backbone into triterpenes and steroids. The head group consists of one or more sugar moieties [26,27,28,29]. Saponins also interact with phospholipid membranes, red blood cells, and cancer cells, as they are able to alter the membrane properties and structure [30,31,32,33,34,35]. The well-known saponin β-aescin is studied here [36,37]. Aescin is an extract from the seeds of the horse chestnut (*Aesculus hippocastanum*) [38] and is a mixture of different saponins with a triterpenic backbone linked via glucuronic acid to two glucose units as the head group (Figure 1c) [39,40]. Aescin on its own forms micelles in aqueous solutions. The critical micelle concentration (cmc) of aescin is 0.33 mM [39]. It has an anti-inflammatory effect, and is used against edema and venous insufficiency [41,42].

The interaction of aescin with DMPC model membranes was intensively studied in dependence on the concentration of the saponin aescin, and a concentration-dependent phase behavior was observed [43]. At low aescin concentrations, the insertion of aescin into the lipid membrane was evidenced, which was accompanied by the lowering of $T_{m}$ and an increase in vesicle size [44]. Increasing the aescin concentration up to its cmc leads to the formation of correlated membrane structures, either intact and aggregated vesicles or larger correlated membrane fragments [31]. At concentrations above the cmc of aescin, the lipid membrane is lastly completely solubilized into bicelles [45]. The whole phase behavior in dependence on aescin concentration and temperature was summarized in a recent review article [43]. In addition to the phase behavior of the DMPC–aescin mixture, the interaction of DMPC with the NSAID ibuprofen and the saponin aescin was also investigated [22]. As has been shown in this work, ibuprofen and aescin interact with the model membrane and change its properties on a molecular level. A lowering of $T_{m}$ was observed.

In comparison to the DMPC–ibuprofen–aescin system, here, the interaction of naproxen and aescin with DMPC model membranes is investigated. Although both mentioned substances are painkillers and cyclo-oxygenase inhibitors, naproxen is used especially for menstrual pain. In their pharmacological properties, they particularly differ in their elimination half-life and thereby their exposure to human cells. Where ibuprofen only has a half-life in the serum of 1.8 h to 2 h, naproxen is significantly more persistent, with a half-life of 16h to 36 h [46,47]. These disparities can be attributed to their different molecular structures. While both 2-arylpropionates contain a stereocenter in the α-position of the propionate group, their molecular backbone differs significantly. Ibuprofen, on the one hand, is based on an alkylated benzene ring. Naproxen, on the other hand, has a larger naphthalene unit with a more polar methoxy group as its molecular backbone. Hence, both NSAIDs differ in size, planarity, and polarity. These important differences render it interesting to study the influence of naproxen on lipid membranes in the presence of aescin, as a different interaction with membranes might be the case. In the human body, naproxen is usually ingested through the intake of medication; for example against menstrual pain, aescin could be contained in certain foods and vein medications. Due to the fact that naproxen is a common analgesic, and aescin is used in vein tablets, it is important to consider the interaction and analyze the influence of the two substances on lipid membranes on a molecular scale [19].

This study was conducted at a physiological pH value of 7.4 in a temperature-dependent manner. We applied the methods of differential scanning calorimetry (DSC), small- and wide-angle X-ray scattering (SAXS, WAXS), and photon correlation spectroscopy (PCS), since their combination is ideally suited for the investigation of various soft-matter and nanoscopic systems [48,49,50]. The influence of naproxen and aescin addition on the DMPC membrane’s $T_{m}$ and the lipid membrane size parameters, derivable from different scattering techniques, is examined.

## 2. Materials and Methods

### 2.1. Chemicals

For vesicle preparation, the phospholipid 1,2-dimyristoyl-*sn*-glycero-3-phosphocholine (DMPC, >99 %, CAS: 18194-24-6, Lipoid GmbH, Ludwigshafen, Germany), the NSAID naproxen (CAS: 22204-53-1, Sigma-Aldrich, Munich, Germany) and the saponin β-aescin (≥95 %, CAS: 6805-41-0, Sigma-Aldrich) were used without further purification. Chloroform (≥99.8 %, CAS: 67-66-3) was purchased from Fisher Chemicals, Waltham, MA, USA. Purified water (arium pro VF System, Sartorius AG, Germany) was used for the aqueous sodium phosphate buffer (50 mM, pH 7.4).

### 2.2. Vesicle Preparation

DMPC (15 mg m$L^{-1}$) and naproxen (10 mol%) were dissolved in chloroform in a round-bottom flask. The molar fraction of naproxen or aescin *w*(naproxen/aescin) was defined according to Equation (1).

(1) $$w\left(\mathrm{naproxen}/\mathrm{aescin}\right)=\frac{n_{\mathrm{naproxen}}/\mathrm{aescin}}{n_{\mathrm{DMPC}}+n_{\mathrm{naproxen}}+n_{\mathrm{aescin}}}$$

After removing the solvent using a rotary evaporator at room temperature in vacuo, the samples were stored overnight in a drying oven (60 ${}^{{}^{\circ}}C$), lastly yielding a thin DMPC–naproxen film, which was rehydrated with different aescin amounts between 0.0 mol% to 1.0 mol% in a phosphate buffer. The vesicles were enlarged with five freeze–thaw-cycles in liquid nitrogen (−196 ${}^{{}^{\circ}}C$) and a water bath (40 ${}^{{}^{\circ}}C$), and then stored frozen at −20 ${}^{{}^{\circ}}C$. The vesicles were extruded at least 15 times at *T* above $T_{m}$ with an extruder (Avanti Polar Lipids Inc., Alabaster, AL, USA, membrane pore size: 50 nm, Whatman) right before performing any measurement.

### 2.3. Differential Scanning Calorimetry (DSC)

DSC experiments were performed by employing a differential scanning heat-flow calorimeter (DSC Q100, TA Instruments, New Castle, DE, USA) to investigate the phase behavior of the lipids in the bilayer. The samples were measured in a hermetically sealed aluminum pan that had been heated up to 40 ${}^{{}^{\circ}}C$ and cooled down to 7 ${}^{{}^{\circ}}C$ with heating and cooling rates of 7 ${}^{{}^{\circ}}C$/min as a pre-equilibration. Afterwards, the sample was heated up to 40 ${}^{{}^{\circ}}C$ with a heating rate of 0.5 ${}^{{}^{\circ}}C$/min. The measured heat flow ΔP was the difference between the heat flow of the sample $P_{S}$ and the reference $P_{0}$ (pan filled with air). The peak maxima of the thermograms, which give $T_{m}$, were determined with Lorentzian fits.

### 2.4. Small- and Wide-Angle X-ray Scattering (SAXS and WAXS)

SAXS and WAXS measurements were performed on the SWING beamline at synchrotron SOLEIL, France. The extruded vesicles were measured with a Linkam-Stage (HFSX350, Linkam Scientific, Salfords, UK) in a flowthrough Kapton^®^ capillary (1 mm, GoodFellow GmbH, Hamburg, Germany) in a temperature range of 10–45 °C in increments of 5 °C. Samples were measured at sample-to-detector distances of 0.5 and 6 m to cover a *q*-range of 0.004Å^−1^ to 1.8Å^−1^. The magnitude of the scattering vector *q* depends on wavelength λ, refractive index *n* (n=1 for X-rays), and scattering angle θ (Equation (2)).

(2) $$q=\frac{4\pi n}{\lambda }\;\cdot \;\sin\left(\frac{\theta }{2}\right)$$

The wavelength was 1.033 Å, and the exposure time was 20 × 1 s. Scattering patterns were detected by an EigerX 4M (Dectris, Baden, Switzerland). The data were evaluated with Foxtrot software (version 3.3.4) [51]. The scattering curves were normalized with respect to intensity, transmission, sample thickness, and exposure time, with the background subtracted and averaged.

By analyzing the position of the peak maximum $q_{\mathrm{peak}}$ of the WAXS curves, information about the changes of the membrane structure regarding the chain–chain correlation distance $d_{\mathrm{WAXS}}$ could be obtained [52]. The peak maximum was precisely determined with a Lorentzian fit and converted into the chain–chain correlation distance applying the following equation (Equation (3)).

(3) $$d_{\mathrm{WAXS}}=\frac{2\pi }{q_{\mathrm{peak}}}$$

The SAXS data were treated with the dynamic rebin formalism in SAXSutilities to improve statistics of individual data points at high *q* values (minimal steps: 1, minimal delta: 0.005) [53]. The SAXS curves were analyzed by using the PCG software package by O. Glatter [54,55]. An indirect Fourier transformation (IFT) was performed with the GIFT program to determine the pair distance distribution function p(r) and the radius of gyration $R_{G}$. The vesicle was divided into the membrane part at low *r*-values and the overall structure at high *r*-values up to $r_{\max}$. At low *r*-values up to 75 Å, 10 cubic B-splines with 10 points per spline were used to approximate the bilayer regime. To describe the vesicular morphology, 15 cubic splines from 75 Å to $r_{\max}$ with 10 points per spline were used. $R_{G}$ was calculated from the integral of the determined p(r) function by Equation (4).

(4) $$R{{}_{G}}^{2}=\frac{\int_{0}^{\infty }p\left(r\right)r^{2}\;dr}{2\int_{0}^{\infty }p\left(r\right)\;dr}$$

Lastly, the modified Kratky–Porod (MKP) analysis was performed to determine the membrane thickness $d_{m,\mathrm{MKP}}$ at $T=10{\;}^{{}^{\circ}}C$ [30,56]. For that, $I\;\cdot \;q^{4}$ was plotted vs. *q* and fitted with a polynomial of fourth order. Then, the maximum was determined, and $d_{m,\mathrm{MKP}}$ was calculated via the relation in Equation (5).

(5) $$d_{m,\mathrm{MKP}}=\frac{2\pi }{q_{\max}}$$

### 2.5. Photon Correlation Spectroscopy (PCS)

The hydrodynamic radius $R_{H}$ of the vesicles was determined via angle-dependent PCS measurements at $T=10{\;}^{{}^{\circ}}C$. Samples were measured in NMR tubes with a 3D LS Spectrometer Pro (LS instruments, Fribourg, Switzerland) equipped with a HeNe Laser (632.8 nm, 1145P; JDSU, Milpitas, CA, USA), decaline index-matching vat, automated goniometer and two detectors (SPCM-AQRH-13-FC, Perkin Elmer, Waltham, MA, USA). The measurements were performed in 3D cross-mode to remove multiple scattering in a scattering angle range of 40${}^{{}^{\circ}}$–110${}^{{}^{\circ}}$ with increments of 5${}^{{}^{\circ}}$ with a measuring time of five times 200 s per angle. The autocorrelation function was generated with a multiple-τ digital correlator and later analyzed by means of inverse Laplacian transformation (CONTIN) to obtain the mean relaxation rate Γ [57,58]. $R_{H}$ was calculated via the Stokes–Einstein equation (Equation (6)) with Boltzmann constant $k_{B}$, temperature *T*, viscosity of the solvent η, and translational diffusion coefficient $D_{T}$. The latter was derived from the slope of the linear dependency of Γ and the squared magnitude of the scattering vector $q^{2}$ (Equation (7)) [59].

(6) $$R_{H}=\frac{k_{B}\;\cdot \;T}{6\;\cdot \;\pi \;\cdot \;\eta \;\cdot \;D_{T}}$$

(7) $$\Gamma =D_{T}\;\cdot \;q^{2}$$

The viscosity of water was calculated in dependence of the temperature [60].

## 3. Results and Discussion

The phospholipid 1,2-dimyristoyl-*sn*-glycero-3-phosphocholine (DMPC) with a constant amount of the nonsteroidal anti-inflammatory drug naproxen of 10 mol% and low amounts of the saponin β-aescin (0.0 mol% to 1.0 mol%) were investigated. In all cases, the aescin concentration was lower than the cmc of aescin [39]. After extrusion, the formation of small and stable particles was revealed through the slight blue opalescence of the samples at temperatures below the main phase transition temperature $T_{m}$. Most likely, these structures were small unilamellar vesicle SUVs. At temperatures above $T_{m}$, the samples turned turbid, and a white precipitate formed that indicated that the vesicles could have begun to aggregate and form larger units. The influence of both additives on the lipid phase behavior, and on the vesicle and membrane structure is analyzed and discussed in the following subsections.

### 3.1. Differential Scanning Calorimetry (DSC): Determination of $T_{m}$

**Figure 2:**
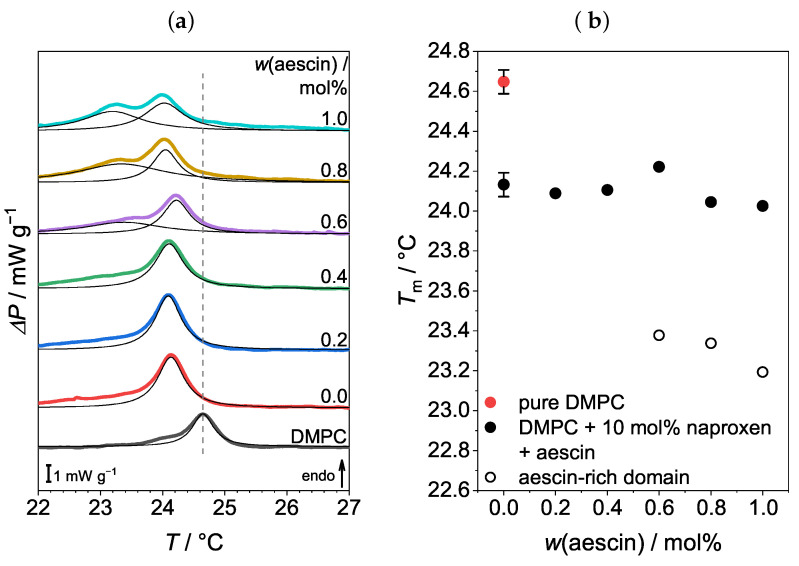
(**a**) Endothermic DSC thermograms of vesicles containing DMPC, 10 mol% naproxen, and different amounts of aescin. The black lines show the Lorentzian fits to determine the maxima of the measured signals. The dashed gray line indicates the $T_{m}$ of pure DMPC vesicles at 24.64±0.06${}^{{}^{\circ}}C$. The addition of naproxen induced a shift of $T_{m}$ to lower temperatures. Aescin amounts from 0.6 mol% and higher led to the appearance of a second peak below $T_{m}$. (**b**) The plot of $T_{m}$ of the DMPC lipid membrane and the aescin-rich domains against *w*(aescin). As an example, the error bars for two data points are given. For the sake of clarity, the other data points were plotted without error bars.

The DMPC SUVs were investigated via differential scanning calorimetry (DSC) in order to resolve the phase transition and determine the $T_{m}$ of the lipid bilayer, at which the temperature-induced conformational change of the hydrocarbon chains from *all-trans* into *gauche* takes place. The endothermic DSC thermograms are shown in Figure 2a. The maximum of the sharp peak gave $T_{m}$ of the bare DMPC SUVs (24.64±0.06 ${}^{{}^{\circ}}C$). This value is in good agreement with $T_{m}$ determined by Sreij et al. [22]. The addition of naproxen led to a significant decrease in the $T_{m}$ of the lipid membrane, as seen from the comparison of the black and red curves in the DSC-thermograms (Figure 2a). The shift in $T_{m}$ due to naproxen incorporation was also visible in data from Manrique-Moreno et al. and resulted from the interaction of the phospholipid headgroup with the hydrophilic part of the drug [15,61]. The increasing amount of aescin led to a further reduction in $T_{m}$, down to 24.00±0.06 ${}^{{}^{\circ}}C$ for an aescin amount of 1 mol%. Above an aescin amount of 0.6 mol%, a shoulder was noticeable that emerged to a broad second peak at temperatures below $T_{m}$. The presence of this additional signal was attributed to the formation of aescin-rich and aescin-poor domains [43]. The $T_{m}$ of the lipid membrane and of the aescin-rich domains was plotted as a function of *w*(aescin) in Figure 2b. The incorporation of additives naproxen and aescin interrupted the ordered structure of the lipid molecules and led to a perturbation in the membrane. Therefore, less energy had to be supplied to complete the phase transition, and $T_{m}$ decreased. The effects described here are already found in the DMPC–ibuprofen–aescin system [22]. Naproxen might not influence the formation of these aescin domains with different phase transition temperatures.

### 3.2. Wide-Angle X-ray Scattering (WAXS): Determination of $d_{\mathrm{WAXS}}$

**Figure 3:**
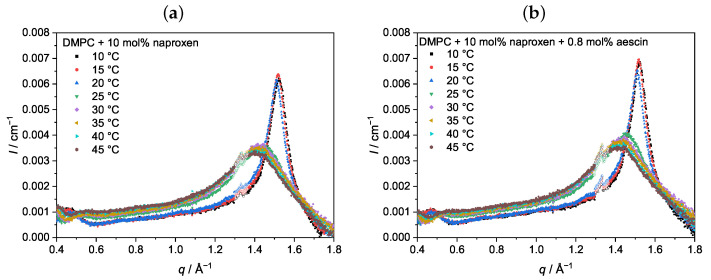
WAXS curves of DMPC vesicles with (**a**) 10 mol% naproxen and (**b**) 10 mol% naproxen and 0.8 mol% aescin at different temperatures. The maximum of the WAXS signal shifted to lower *q*-values with increasing temperature. The open circles around 1.3 Å^−1^ mark artifacts that were not considered for evaluation.

The vesicles containing DMPC, naproxen, and aescin were analyzed with wide-angle X-ray scattering (WAXS). The temperature-dependent scattering curves of DMPC with 10 mol% naproxen and 0.0 mol% and 0.8 mol% aescin are exemplarily shown in Figure 3. The WAXS measurements were performed at a temperature range of 10–45 °C in increments of 5 ${}^{{}^{\circ}}C$. The WAXS curves of samples with other *w*(aescin) looked similar and all are presented in the Supplementary Information (Figures S1 and S2). The open circles at around 1.3 Å^−1^ mark artifacts that occurred in both the sample and the background measurement, and were not considered in the analysis. Due to thermally induced aggregation, scattering curves for DMPC with naproxen and 1 mol% aescin are only shown up to 25 ${}^{{}^{\circ}}C$ (Figure S2). The WAXS signals below $T_{m}$ showed a typical narrow shape, whereas the signal broadened in the L${}_{\alpha }$ phase above $T_{m}$ [62].

**Figure 4:**
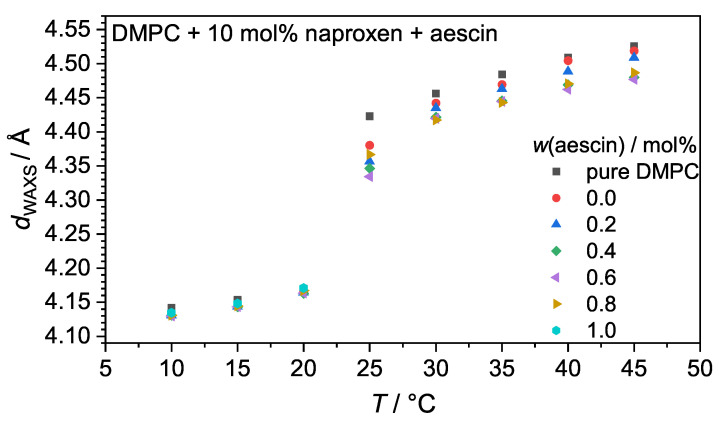
Temperature-dependent $d_{\mathrm{WAXS}}$ values of the DMPC vesicles with naproxen and aescin, calculated from the peak maximum $q_{\mathrm{peak}}$ via Equation (3).

The peak maximum $q_{\mathrm{peak}}$ shifted with increasing temperature to lower *q*-values and changed from a sharp, narrow peak below $T_{m}$ into a broadened peak above $T_{m}$. Additionally, the most significant change in $q_{\mathrm{peak}}$ was visible around $T_{m}$. The same trend had been observed by Sreij et al. in small unilamellar DMPC vesicles with ibuprofen and aescin [22]. $d_{\mathrm{WAXS}}$ was determined by Equation (3) from $q_{\mathrm{peak}}$ and is plotted in Figure 4. Due to the conformational change of the lipids in the bilayer during the main phase transition, $d_{\mathrm{WAXS}}$ is a temperature-dependent parameter. At temperatures below $T_{m}$, $d_{\mathrm{WAXS}}$ was the smallest, as the lipid DMPC was in the L${}_{\beta^{\prime }}$ phase where the chains were packed in a hexagonal lattice [13]. An increase in temperature up to $T_{m}$ resulted in a slight increase in $d_{\mathrm{WAXS}}$ due to thermal expansion. A comparison of the samples with and without naproxen and aescin shows that the incorporation of both did not impact $d_{\mathrm{WAXS}}$ as long as $T<T_{m}$.

Upon an increase of *T* above $T_{m}$, the lipid underwent the main phase transition into the L${}_{\alpha }$ phase, in which $d_{\mathrm{WAXS}}$ is increased due to the more flexible rearrangement of the lipid molecules. Compared to pure DMPC vesicles, $d_{\mathrm{WAXS}}$ slightly decreased if 10 mol% naproxen was added to the DMPC sample. A further decrease upon aescin addition and with increasing *w*(aescin) was observed, additionally. The accumulation of aescin into the DMPC membrane, which could be deduced from DSC, led to a compression of the DMPC molecules, which was expressed in a smaller $d_{\mathrm{WAXS}}$ value. Hence, the DMPC membrane in the L${}_{\alpha }$ phase was stabilized in the presence of aescin.

### 3.3. Analysis by Small-Angle X-ray Scattering (SAXS)

**Figure 5:**
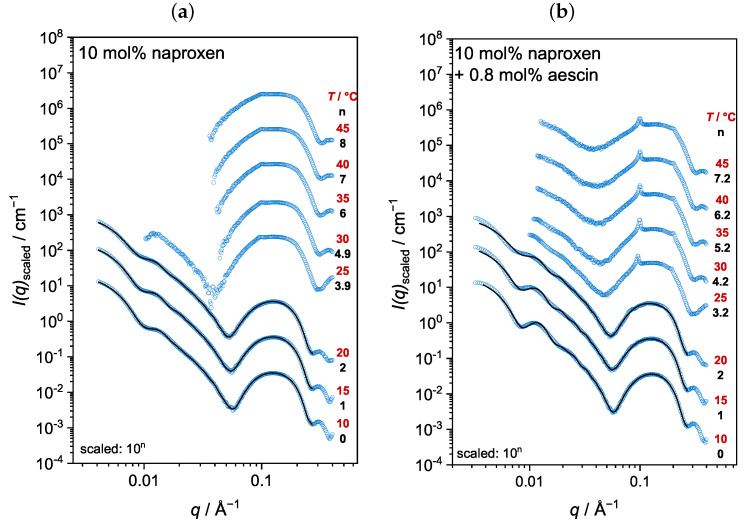
SAXS curves of (**a**) DMPC vesicles with 10 mol% naproxen and (**b**) vesicles with 10 mol% naproxen and 0.8 mol% aescin at different temperatures (see red numbers on the right). Scattering curves were scaled by powers of 10 for better visibility. Solid lines are IFT approximations to the scattering data.

Small-angle X-ray scattering (SAXS) measurements of the DMPC–naproxen–aescin samples were performed at the SWING beamline of the Soleil synchrotron. Datasets for DMPC vesicles containing naproxen and DMPC vesicles containing naproxen and 0.8 mol% aescin are shown exemplarily in Figure 5. SAXS data for all samples are presented in the Supplementary Information (Figure S3). Scattering data of the reference system, pure DMPC, are shown in Figure S3a. Pure DMPC vesicles were stable in the investigated temperature range. Below and above $T{}_{m}$, the formation of aggregated structures was not observed (Figure S3 and Figure 9).

Below $T_{m}$, the scattering curves were, in the two cases of DMPC with 10 mol% naproxen (Figure 5a), and DMPC with 10 mol% naproxen and 0.8 mol% aescin (Figure 5b), characteristic for unilamellar vesicles [30]. The oscillations at low *q*-values indicate a rather narrow distribution of the vesicle size, and the signal at around 0.1 Å^−1^ represents the form factor of the lipid membrane. When $T_{m}$ was reached, the lipid membrane underwent a conformational change from the $L_{\beta }^{\prime }$ into the $L_{\alpha }$ phase (at $T\geq 25{\;}^{{}^{\circ}}C$). Hence, the continuous DMPC membrane lost its dense packing, and penetration of water molecules into the lipid membrane was eased [63]. As a consequence of that, the electron density difference between the membrane and the solvent decreased, so that a size resolution of the vesicle structure was not possible anymore due to the decreased scattering intensity at low *q*. At the high *q*-region, additionally to the membrane form factor, a structure factor appeared if an additive was present in the sample. If only naproxen had been added to the DMPC, the correlation signal at 0.1 Å^−1^ was very weak, but became more intense if the aescin content was also increased. This indicates the formation of correlated bilayer structures induced by the presence of aescin. Additionally, a second signal $q_{2}$ at around 0.2 Å^−1^ appeared and, like the signal $q_{1}$ at 0.1 Å^−1^, increased in intensity with increasing *w*(aescin). From the ratio of both signals ($2q_{1}\approx q_{2}$) it can be concluded that a lamellar-phase-like arrangement was present in the sample.

In the following, first, the pair distance distribution function p(r) and the radius of gyration $R_{G}$ are determined with indirect Fourier transformation (IFT) for all samples at $T<T_{m}$. Afterwards, the membrane thickness $d_{m,\mathrm{MKP}}$ is calculated by the modified Kratky–Porod (MKP) method for all samples at $T=10{\;}^{{}^{\circ}}C$. Lastly, we focus on the membrane aggregation at $T>T_{m}$.

#### 3.3.1. Pair Distance Distribution Function p(r) and Radius of Gyration $R_{G}$ by Indirect Fourier Transformation (IFT) at $T<T_{m}$

**Figure 6:**
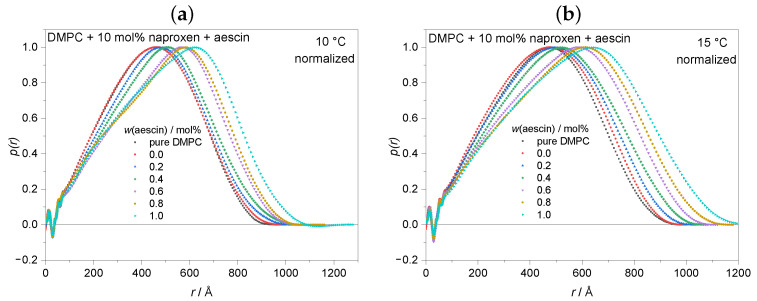

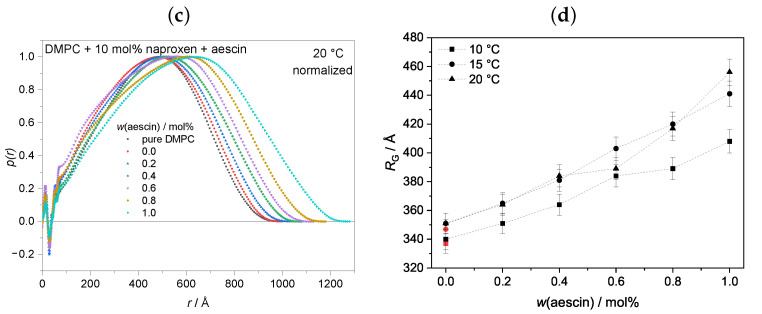
Pair distance distribution functions p(r) of pure DMPC vesicles and vesicles containing naproxen and aescin at (**a**) 10 ${}^{{}^{\circ}}C$, (**b**) 15 ${}^{{}^{\circ}}C$ and (**c**) 20 ${}^{{}^{\circ}}C$. Functions were normalized to the maximum of p(r) for better comparability. (**d**) Radius of gyration $R_{G}$ of DMPC vesicles containing naproxen and different amounts of aescin against *w*(aescin) at $T=10{\;}^{{}^{\circ}}C,15{\;}^{{}^{\circ}}C$ and 20 ${}^{{}^{\circ}}C$. The dashed lines are only visual guides. The red symbols depict $R_{G}$ of pure DMPC vesicles.

The indirect Fourier transformation (IFT) method is a model-independent tool to determine the pair distance distribution function (PDDF or p(r)) and thereby the radius of gyration $R_{G}$ of a scattering particle [54,55]. Only samples recorded at $T<T_{m}$ were considered for evaluation because, for these samples, the overall size and structure were accessible from the low *q*-range. The normalized p(r) functions at temperatures of 10 ${}^{{}^{\circ}}C$, 15 ${}^{{}^{\circ}}C$ and 20 ${}^{{}^{\circ}}C$ are shown in Figure 6a–c. Oscillation at low distances *r* is a typical characteristic of a lipid membrane with head-tail contrast that occurs due to the difference in the electron densities of the lipids head and tail parts within the lipid bilayer [64]. The maximum of the p(r) functions shifted to higher distances with increasing *w*(aescin). This indicates that the vesicles grew.

$R_{G}$, obtained by the p(r) function from Equation (4), was plotted as a function of *w*(aescin) at 10 ${}^{{}^{\circ}}C$, 15 ${}^{{}^{\circ}}C$ and 20 ${}^{{}^{\circ}}C$ in Figure 6d. The addition of naproxen to the DMPC vesicles showed no significant impact on $R_{G}$ at a constant temperature. DSC clearly showed the incorporation of naproxen into the lipid membrane, but this incorporation seems not to influence the $R_{G}$ of the vesicle. However, $R_{G}$ increased with the addition of aescin and increasing *w*(aescin), which indicates successful incorporation of naproxen and aescin into the lipid membrane. At constant *w*(aescin) but increasing temperature, an enlargement of the vesicle size was observed due to the prephase change from L${}_{\beta }$ into P${}_{\beta^{\prime }}$, which had also been seen by Sreij et al. [31].

#### 3.3.2. Membrane Thickness $d_{m,\mathrm{MKP}}$ by Modified Kratky Porod (MKP) at $T<T_{m}$

**Figure 7:**
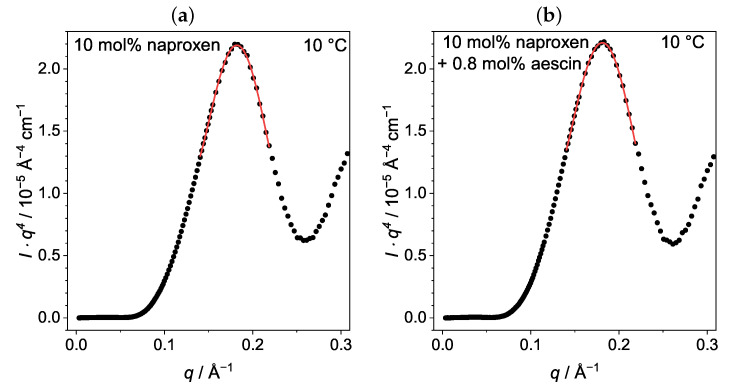
MKP plots for DMPC vesicles (**a**) with 10 mol% naproxen and (**b**) with 10 mol% naproxen and 0.8 mol% aescin at $T=10{\;}^{{}^{\circ}}C$. Red lines are fourth-order polynomial fits.

**Figure 8:**
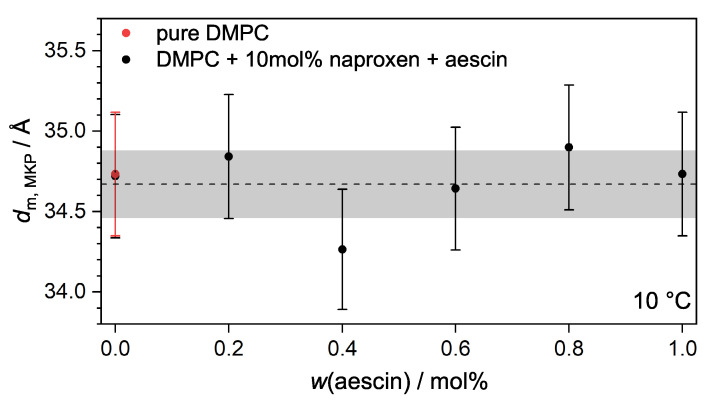
Membrane thicknesses $d_{m,\mathrm{MKP}}$ of vesicles determined via MKP approximation at $T\;=\;10{\;}^{{}^{\circ}}C$ in dependence of *w*(aescin). The dashed line gives the mean value of $d_{m,\mathrm{MKP}}$ at 34.67±0.21Å. The red circle depicts the membrane thickness of pure DMPC vesicles.

The membrane thickness $d_{m,\mathrm{MKP}}$ of nonaggregating vesicles can be analyzed with the model-independent modified Kratky–Porod (MKP) method [18,30]. Aggregation effects could be excluded for samples up to 0.4 mol% aescin for $T<T_{m}$, but samples from 0.6 mol% aescin showed an indication of aggregation at $T=15{\;}^{{}^{\circ}}C$. Therefore, only data recorded at 10 ${}^{{}^{\circ}}C$ were considered for evaluation here. On the basis of SAXS data, $I\left(q\right)\;\cdot \;q^{4}$ was plotted against *q*. Examples for DMPC vesicles containing naproxen, and vesicles with naproxen and 0.8mol% aescin are shown in Figure 7. The resulting plots were fitted with a fourth-order polynomial to obtain the maximal *q*-value $q_{\max}$ that was used to calculate $d_{m,\mathrm{MKP}}$ according to Equation (5). The calculated $d_{m,\mathrm{MKP}}$ values were plotted as a function of *w*(aescin) at $T=10{\;}^{{}^{\circ}}C$ (Figure 8).

The bilayer thickness of pure DMPC vesicles at $T=10{\;}^{{}^{\circ}}C$ was 34.72±0.38 Å, which nicely corresponded to the value obtained by Sreij et al. [30], also determined for bare DMPC SUVs. Within the accuracy of the method, no significant change in $d_{m,\mathrm{MKP}}$ was observed upon the addition of naproxen or upon the increase in *w*(aescin). Therefore, the incorporation of both additives had no influence on the thickness of the DMPC membrane. An explanation might be that aescin was filling gaps in the membrane that had been created by the incorporation of naproxen.

#### 3.3.3. Membrane Structure at $T>T_{m}$

**Figure 9:**
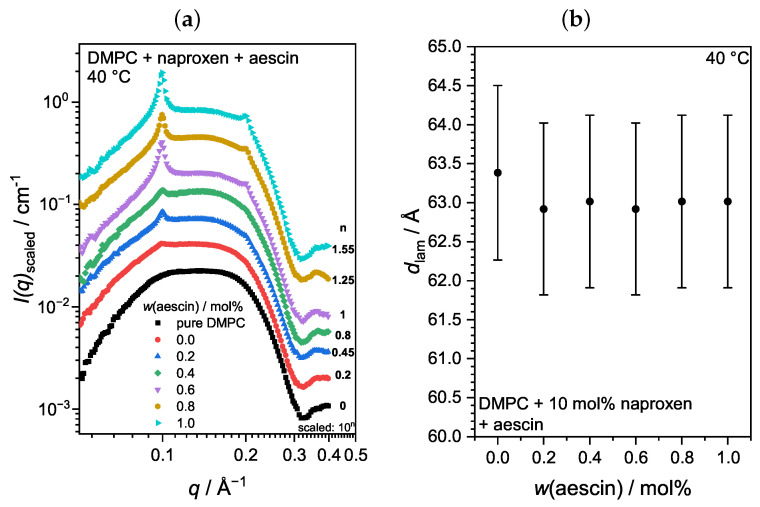
(**a**) SAXS curves of DMPC vesicles with 10mol% naproxen and varying amounts of aescin at $T=40{\;}^{{}^{\circ}}C$. Scattering curves are scaled by powers of 10 for better visibility. (**b**) Lamellar repeat distance $d_{\mathrm{lam}}$ of the correlated bilayers calculated from the maximum of the first order Bragg peak at $T=40{\;}^{{}^{\circ}}C$.

Increasing the temperature above $T_{m}$ caused the lipid membrane to undergo a structural transition from the P${}_{\beta^{\prime }}$ into the L${}_{\alpha }$ phase. A higher space requirement of the single DMPC molecules accompanied this transition (compare Figure 4). The membrane itself became less dense, which caused the contrast of the overall vesicle to decrease. The size and structural determination from the low *q*-region was not possible anymore because no significantly differing data from the scattering background could be recorded. However, information about the membrane structure itself was still accessible. Representatively for all $T>T_{m}$, the scattering data at 40 ${}^{{}^{\circ}}C$ are shown in Figure 9a for all samples investigated. For samples containing aescin at $T>T_{m}$, i.e., from 30 ${}^{{}^{\circ}}C$ onwards, two additional peaks in the bilayer region at around 0.1 Å^−1^ as first-order and at around 0.2 Å^−1^ as a second-order signal appeared. These signals, resulting from the formation of correlated membrane structures, became more intense with increasing *w*(aescin). The ratio between the signal positions ($2q_{1}\approx q_{2}$) indicates the formation of a lamellar structure, and the rising peak intensity indicates the formation of a higher number of correlated bilayers within this lamellar arrangement.

The repeat distance of the correlated lamellae $d_{\mathrm{lam}}$ could be calculated from the position $q_{1}$ of the first order correlation signal, located at around 0.1 Å^−1^, with the relation $d_{\mathrm{lam}}=2\pi /q_{1}$. This distance comprises an entire bilayer and an interstitial water layer. Figure 9b depicts $d_{\mathrm{lam}}$ as a function of *w*(aescin). For aescin-containing samples, $d_{\mathrm{lam}}$ remained constant, which indicates that a further addition of aescin had no influence on the correlation distance of the lamellar stack. For the system containing only DMPC and naproxen, a higher repeat distance was observed, which might have been an artifact from the weak and quite broad signal.

For the samples containing 0.6 mol% or more of aescin, correlation signals in the membrane region were even visible at temperatures below $T_{m}$ (see Figure S3). In contrast to $T>T_{m}$, three distinct peaks appeared, indicating the formation of different structures with different spacing. The SAXS curves of all samples at $T=20{\;}^{{}^{\circ}}C$ are shown in Figure S4 in the Supplementary Information. This is in analogy to the naproxen-free system, in which these kinds of signals were observed for aescin contents of 1 mol% to 5 mol% and at temperatures around $T_{m}$ [31]. The correlated membrane structures can be interpreted as precursors of solubilized membrane fragments and finally completely decomposed bicelles. Therefore, the present observation gives a hint towards the increased ability of aescin to decompose a lipid vesicle membrane in presence of naproxen [31,43]. This behavior is analogous to the previously studied ibuprofen–DMPC system with added aescin [22].

### 3.4. Photon Correlation Spectroscopy (PCS): Determination of $R_{H}$ below $T_{m}$

**Figure 10:**
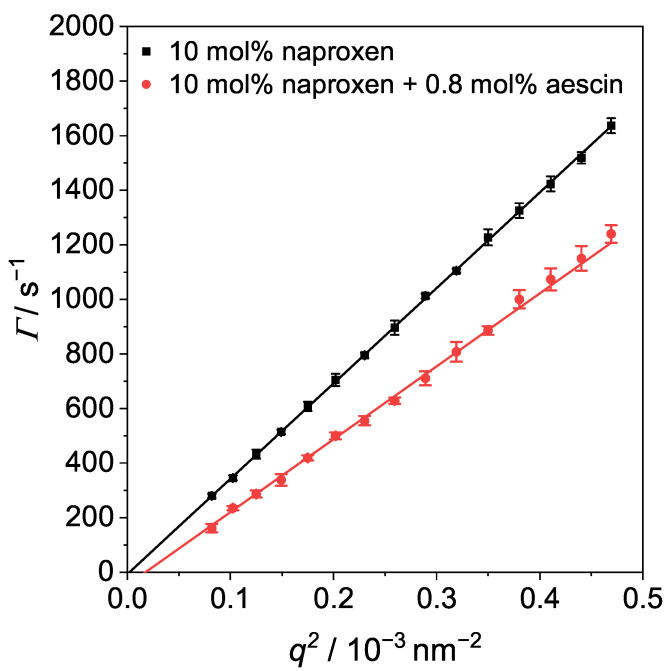
Γ plotted against $q^{2}$ measured by angle-dependent PCS at $T=10{\;}^{{}^{\circ}}C$ for DMPC vesicles with 10 mol% naproxen and vesicles with 10 mol% naproxen and 0.8 mol% aescin. Black and red lines are linear fits to determine $D_{T}$.

To avoid multiple scattering effects, the vesicles were measured in a 3D photon cross-correlation spectroscopy (3D-PCS) setup at different angles at $T=10{\;}^{{}^{\circ}}C$ to determine the hydrodynamic radius $R_{H}$ below $T_{m}$. Moreover, at low *T*, the formation of aggregates is mostly avoided. The plots of relaxation rate Γ for DMPC vesicles with naproxen and vesicles with naproxen and 0.8 mol% aescin are shown in Figure 10. In addition, the plots for the other samples are shown in the Supplementary Information (Figures S5 and S6).

**Figure 11:**
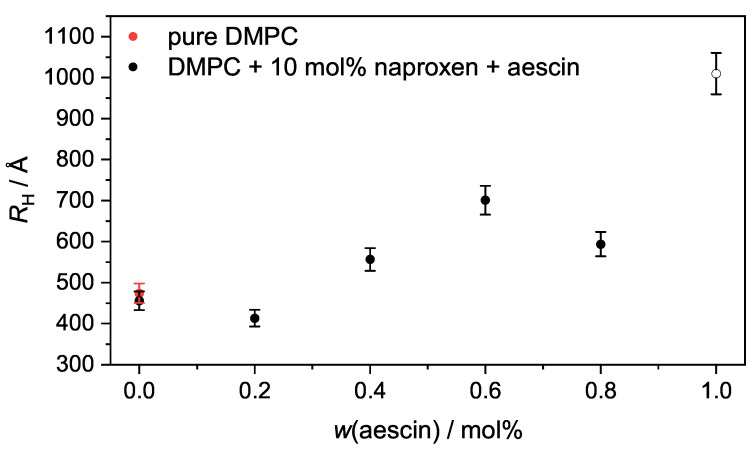
$R_{H}$ at $T=10{\;}^{{}^{\circ}}C$ of the DMPC vesicles containing 10 mol% naproxen and aescin is plotted as a function of *w*(aescin). The red circle depicts the hydrodynamic radius of pure DMPC vesicles.

Via Equation (6), $R_{H}$ can be calculated. The hydrodynamic radius was plotted as a function of *w*(aescin) in Figure 11. Pure DMPC vesicles have a $R_{H}$ of (474±24) Å at $T=10{\;}^{{}^{\circ}}C$. $R_{H}$ remained constant upon addition of naproxen and increased with increasing *w*(aescin) from around 450Å–1010Å, which indicates the successful incorporation of aescin molecules into the lipid membrane. This trend was also shown by Sreij et al. [44]. $R_{H}$ was larger than $R_{G}$ because the hydrodynamic radius includes a hydration shell that is enlarged compared to normal lipids due to the presence of the sugar moieties. A comparison of the two radii is shown in Figure S7. The values of $R_{H}$ and PDI are given in Table S1. Both radii increased with increasing *w*(aescin). The high apparent $R_{H}$ for the DMPC vesicles containing naproxen and 1.0 mol% aescin (open circle (Figure 11)) might have been causedby aggregation of the vesicles and was not considered.

## 4. Conclusions

**Figure 12:**
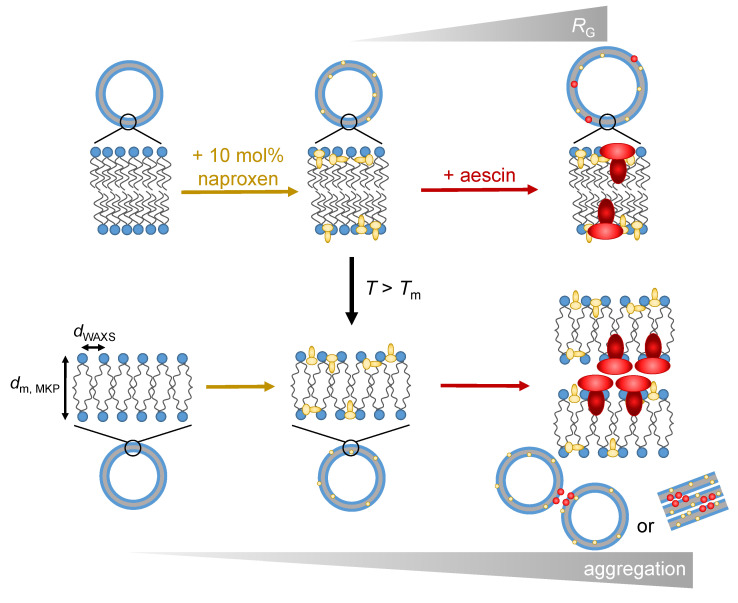
Schematic illustration of the proposed interplay of the drug naproxen and the saponin aescin with DMPC vesicles as model membranes. The figure displays the intercalation of both substances into the membranes and the aescin-induced formation of aggregates.

In this work, we analyzed DMPC model membranes containing 10 mol% of the NSAID naproxen and different amounts of the natural biosurfactant aescin (0.0 mol% to 1.0 mol%) with DSC, WAXS, SAXS, and PCS in a temperature-dependent manner. The obtained results confirm the interactions of both naproxen and aescin with the lipid bilayer formed by DMPC, independent of the method used. The obtained results were schematically summarized (see Figure 12), showing a possible scenario of the arrangement of the two additives in the lipid membrane. The intercalation of naproxen and aescin into the membrane was a priori assumed according to the publications by Alsop et al., and Sreij et al., and was evidenced with the experimental results of DSC and WAXS in this study [22,23]. The incorporation of naproxen into the DMPC membrane resulted in a lowered $T_{m}$. The addition of aescin led to a further decrease in $T_{m}$, and the formation of aescin-rich domains became visible from DSC. $d_{\mathrm{WAXS}}$ of the DMPC molecules increased with rising temperature, with the most significant change happening when passing $T_{m}$. At $T<T_{m}$, the addition of neither naproxen nor aescin showed a significant influence on $d_{\mathrm{WAXS}}$. Above $T_{m}$, naproxen addition induced a decrease of $d_{\mathrm{WAXS}}$ and with increasing *w*(aescin), $d_{\mathrm{WAXS}}$ was even further reduced.

At all temperatures below $T_{m}$, well-defined small unilamellar vesicles were present, which was proven via SAXS. The membrane thickness was 34.7 Å through the MKP method at $T=10{\;}^{{}^{\circ}}C$ and did not change significantly after the addition of either naproxen or aescin addition. Moreover, the addition of naproxen had no influence on $R_{G}$; the addition of aescin, however, resulted in an increase in $R_{G}$. The vesicles tended to aggregate at $T>T_{m}$, which was revealed by the lamellar correlation peak. The lamellar repeat distance was constant for all aescin amounts, but the number of correlated bilayers seemed to grow by increasing *w*(aescin). This indicates that aescin acts as a linker between the two different bilayers. Moreover, the vesicles at $T=10{\;}^{{}^{\circ}}C$ showed a low polydispersity measured by PCS. $R_{H}$ was increased by the addition of aescin.

With these measurements, both the NSAID naproxen and the saponin aescin were incorporated into the DMPC model membrane. Due to the interactions between naproxen and aescin molecules with the DMPC molecules, the membrane properties were changed. Despite the discussed differences of ibuprofen and naproxen, their effects on the DMPC model membranes were comparable. The employed methods did not resolve the type and strength of these interactions, but showed their effects on the vesicle properties. The molecular structures (see Figure 1) suggest the presence of predominantly electrostatic and hydrophobic forces [24]. A detailed analysis of the type and strength of the present forces is beyond the scope of this paper, but could be studied by methods such as avoided level crossing muon spin resonance (ALC-*μ*SR) to gain an insight on the orientation of additives due to the orienting effect of the electric field of the membrane [65,66,67]. In addition, theoretical simulations such as those by Selyutina et al. with DPPC and glycyrrhizin would be interesting to understand the DMPC-aescin-naproxen system on a more detailed level [68]. It would be very interesting to address the question of whether aescin enhances or decreases the activity of naproxen. Another scattering technique that can be used to obtain more information about the lipid bilayer and the lamellar structures is small-angle neutron scattering (SANS) [69]. Moreover, it would be a benefit to investigate these vesicles via neutron spin echo spectroscopy to obtain deeper knowledge about the fluidity and stiffness of the membrane at different phase states in the presence of naproxen and aescin [30,70]. With these results, the effect of NSAIDs and saponins on biomembranes can be better estimated for pharmaceutical applications. This understanding is of interest to expand the knowledge of interactions between biological substances and membranes. In the future, it is necessary to correlate the results obtained in this study with pharmacological studies.

## Data Availability

The data are available on request from the corresponding author.

## Supplementary Materials

The following supporting information can be downloaded at: https://www.mdpi.com/article/10.3390/pharmaceutics15020379/s1, Figure S1. Temperature-dependent WAXS-curves for (a) pure DMPC, (b) DMPC with 10 mol% naproxen and (c–f) different amounts of aescin, ranging from 0.2 mol% to 0.8 mol%. The open circles mark artifacts that are not considered for evaluation. Figure S2. Temperature-dependentWAXS-curves for DMPC vesicles with 10 mol% naproxen and 1.0 mol% aescin. The open circles mark artifacts that are not considered for evaluation. Figure S3. Temperature-dependent SAXS-curves for (a) pure DMPC, (b) DMPC with 10 mol% naproxen and (c–f) different amounts of aescin, ranging from 0.2 mol% to 1.0 mol%. Curves are scaled by multiples of 10. Solid lines are IFT approximations. Figure S4. SAXS-curves of DMPC vesicles with 10 mol% naproxen and varying amounts of aescin at *T* = 20 °C. Scattering curves are scaled by powers of 10 for better visibility. Figure S5. Γ plotted against *q*${}^{2}$ measured by angle-dependent PCS at *T* = 10 °C for (a) pure DMPC, (b) DMPC with 10 mol% naproxen and (c–f) different amounts of aescin, ranging from 0.2 mol% to 0.8 mol%. Red lines are linear fits to determine *D*_T_. Figure S6. Γ plotted against *q*${}^{2}$ measured by angle-dependent PCS at *T* = 10 °C for DMPC with 10 mol% naproxen and 1.0 mol% aescin. Red lines are linear fits to determine *D*_T_. Table S1. Hydrodynamic radius *R_H_* and polydispersity index PDI obtained from CONTIN evaluation of PCS data in dependence on the aescin amount *w*(aescin). Data was recorded at *T* = 10 °C. Figure S7. Comparison of *R_G_* and *R_H_* values, both measured at *T* = 10 °C and in dependence on *w*(aescin). The red symbols mark the values for pure DMPC vesicles.
