# Supplementary material for: Interactions between DMPC Model Membranes, the Drug Naproxen, and the Saponin β-Aescin
Source: Pharmaceutics. 2023 Jan 22;15(2):379. doi: 10.3390/pharmaceutics15020379 (PMC9960855; doi:10.3390/pharmaceutics15020379)
Supplement: Supplementary file 1 [file pharmaceutics-15-00379-s001.zip › pharmaceutics-2075043-supplementary.pdf]

Supplementary Materials: Interactions between DMPC model membranes, the drug naproxen, and the saponin β-aescin

Pia Hägerbäumer [1], Friederike Gräbitz-Bräuer [1], Marco Annegarn [1], Carina Dargel [1,2], Tim Julian Stank [1], Thomas Bizien [3], and Thomas Hellweg [1,*]

1. WAXS

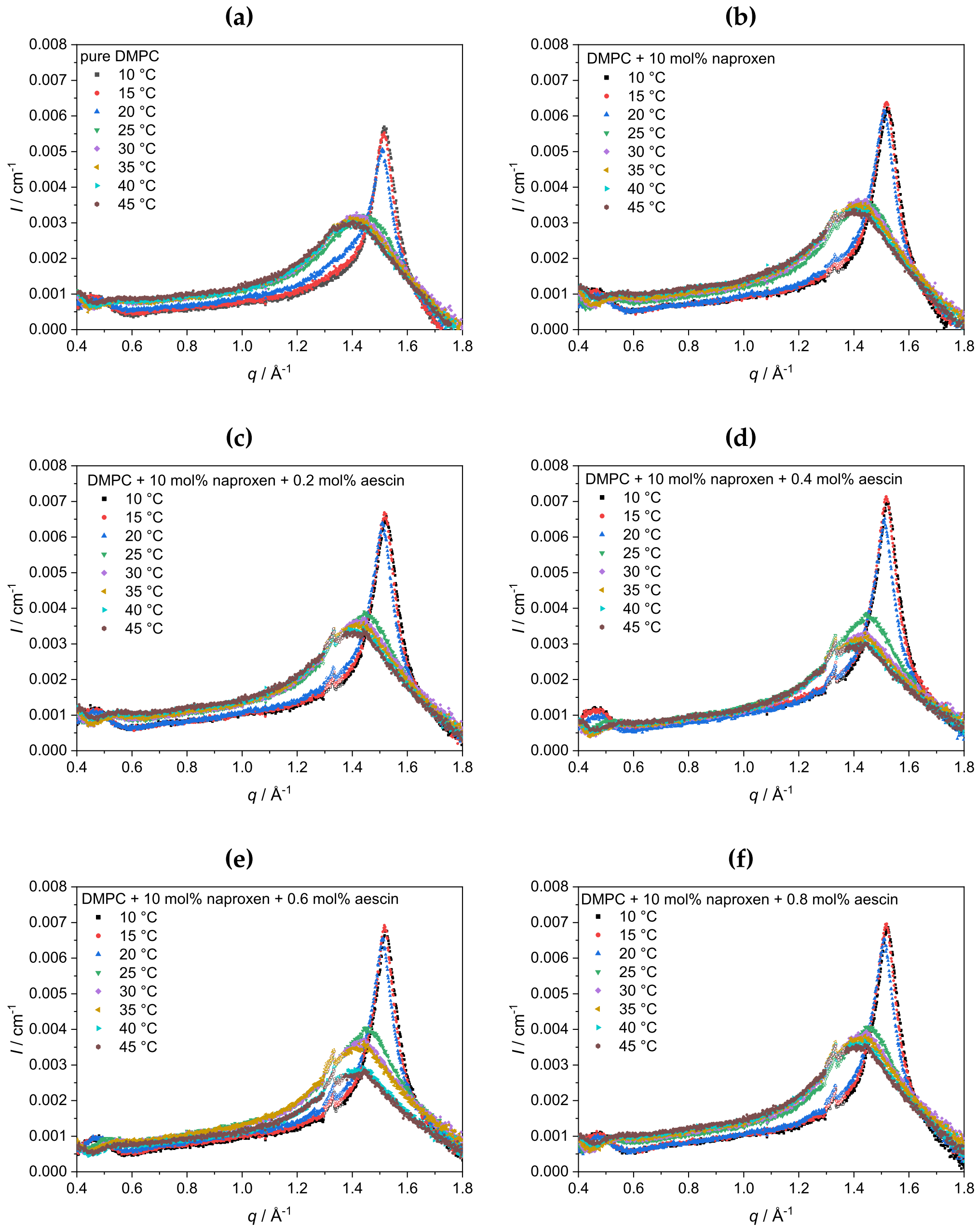

Figure S1. Temperature-dependent WAXS-curves for (a) pure DMPC, (b) DMPC with 10 mol% naproxen and (c) - (f) different amounts of aescin, ranging from 0.2 mol% to 0.8 mol%. The open circles mark artifacts that are not considered for evaluation.

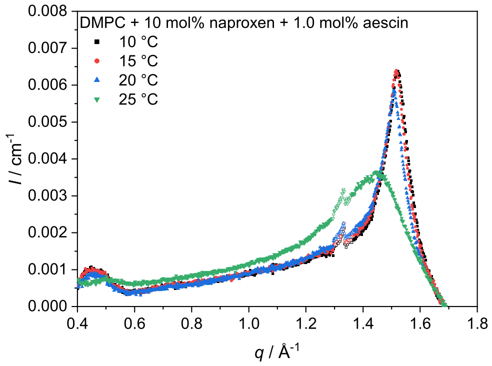

Figure S2. Temperature-dependent WAXS-curves for DMPC vesicles with 10 mol% naproxen and 1.0 mol% aescin. The open circles mark artifacts that are not considered for evaluation.

2. SAXS

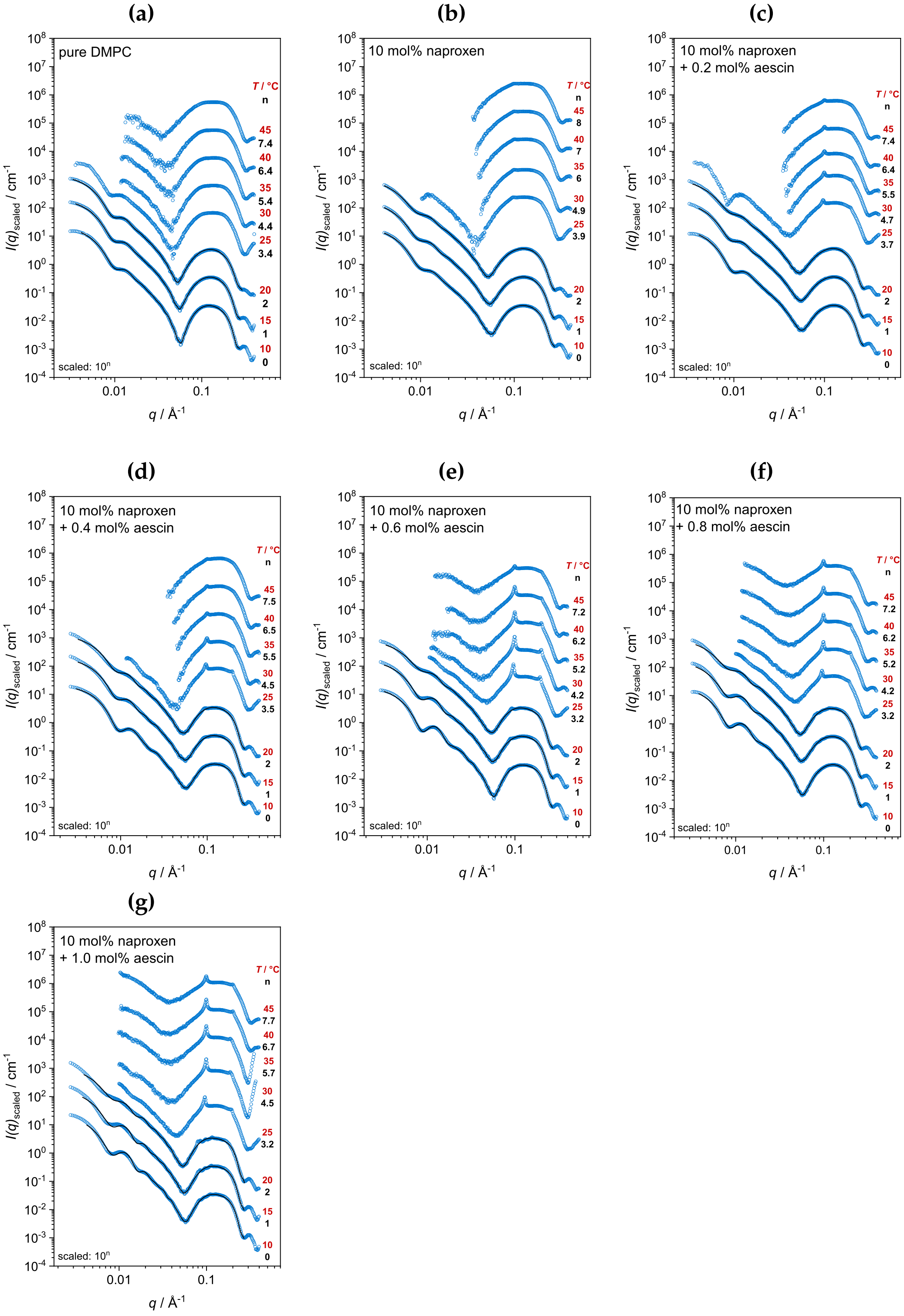

Figure S3. Temperature-dependent SAXS-curves for (a) pure DMPC, (b) DMPC with 10 mol% naproxen and (c) - (f) different amounts of aescin, ranging from 0.2 mol% to 1.0 mol%. Curves are scaled by multiples of 10. Solid lines are IFT approximations.

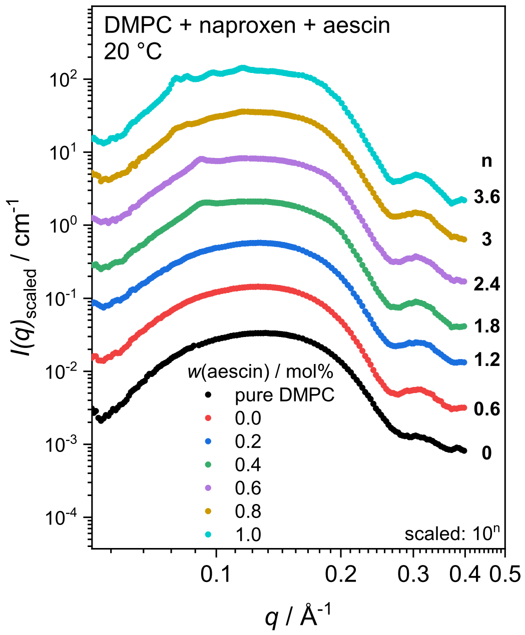

Figure S4. SAXS-curves of DMPC vesicles with 10 mol% naproxen and varying amounts of aescin at $T = 20\,^{\circ}\mathrm{C}$. Scattering curves are scaled by powers of 10 for better visibility.

3. PCS

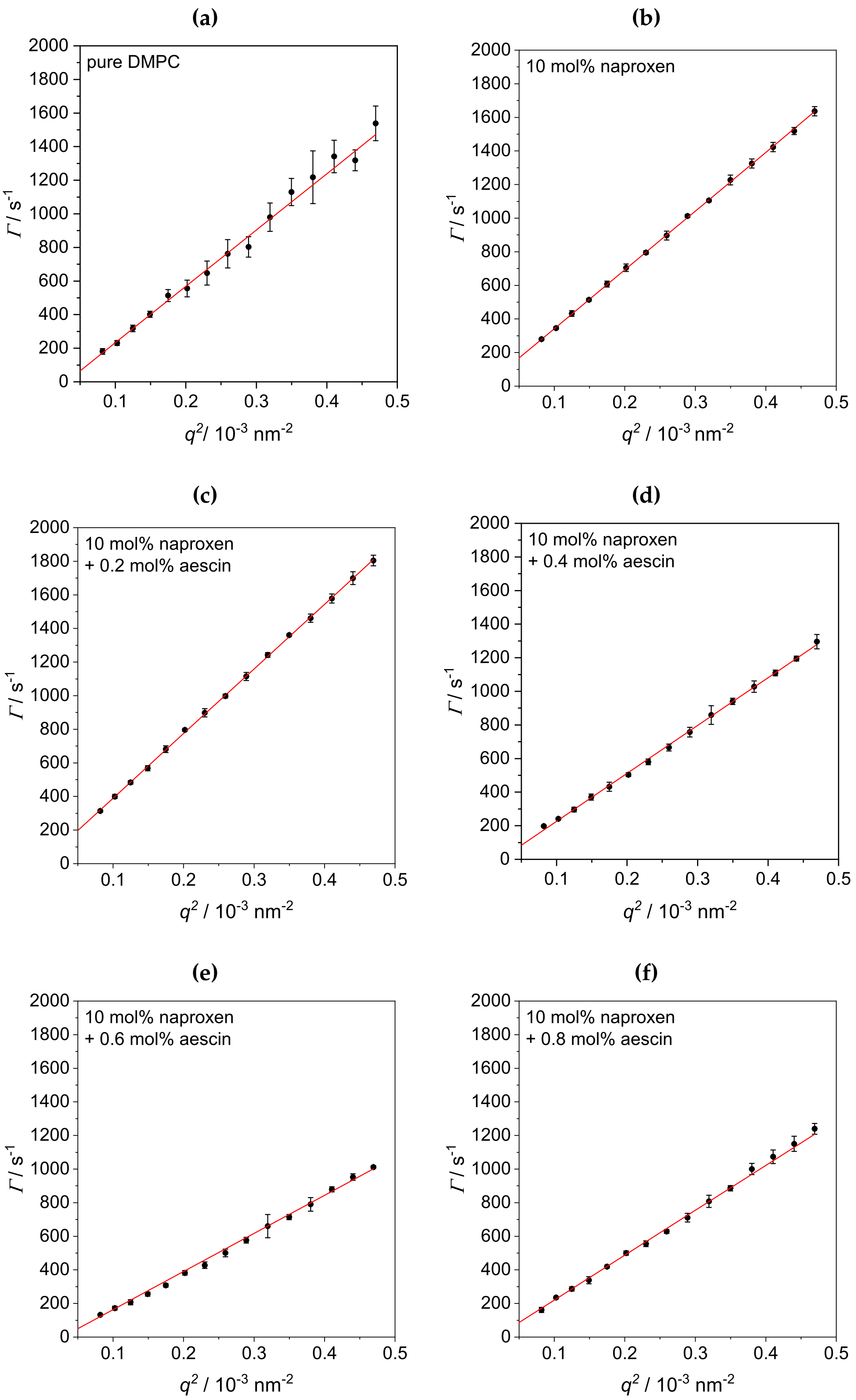

Figure S5. Γ plotted against q^2 measured by angle-dependent PCS at $T = 10\,°C$ for (a) pure DMPC, (b) DMPC with 10 mol% naproxen and (c) - (f) different amounts of aescin, ranging from 0.2 mol% to 0.8 mol%. Red lines are linear fits to determine D_T.

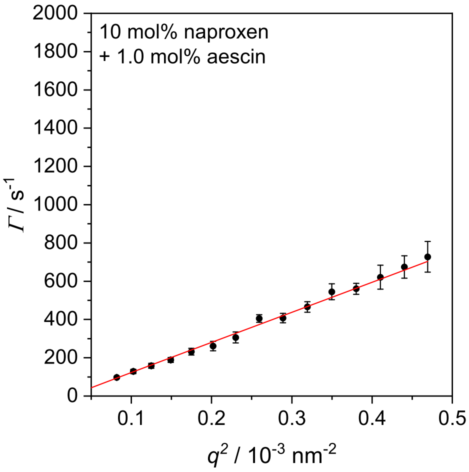

Figure S6. Γ plotted against q^2 measured by angle-dependent PCS at $T = 10\,^\circ$C for DMPC with 10 mol% naproxen and 1.0 mol% aescin. Red lines are linear fits to determine D_T.

Table S1. Hydrodynamic radius R_H and polydispersity index PDI obtained from contin evaluation of PCS data in dependence on the aescin amount w(aescin). Data was recorded at $T = 10\,^\circ$C.

w(aescin) / mol%	R_H / Å	PDI
pure DMPC	474 ± 24	0.29
0.0	456 ± 23	0.06
0.2	413 ± 21	0.09
0.4	557 ± 28	0.31
0.6	701 ± 35	0.35
0.8	594 ± 30	0.28
1.0	1010 ± 50	0.33

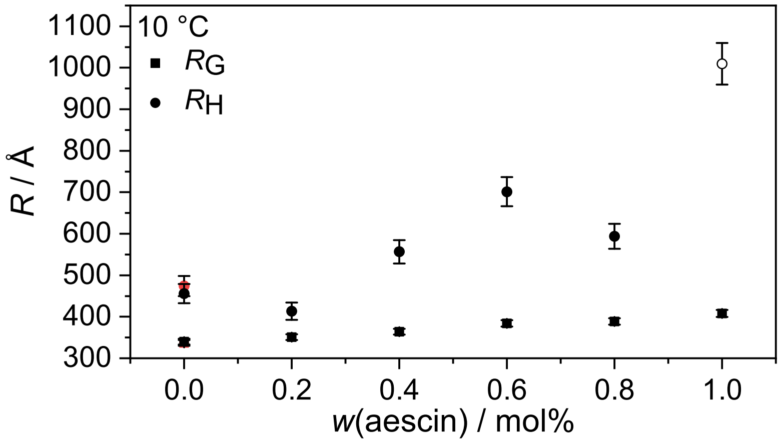

Figure S7. Comparison of R_G and R_H values, both measured at $T = 10\,^\circ$C and in dependence on w(aescin). The red symbols mark the values for pure DMPC vesicles.
